# Impact of Swabbing Location, Self-Swabbing, and Food Intake on SARS-CoV-2 RNA Detection

**DOI:** 10.3390/microorganisms12030591

**Published:** 2024-03-15

**Authors:** Sarah Dräger, Flavio Bruni, Melina Bernasconi, Anya Hammann-Hänni, Vlastimil Jirasko, Alexander Tanno, Yves Blickenstorfer, Karoline Leuzinger, Hans H. Hirsch, Michael Osthoff

**Affiliations:** 1 Division of Internal Medicine, University Hospital Basel, 4031 Basel, Switzerland; 2 Department of Clinical Research, University of Basel, 4031 Basel, Switzerland; 3 Laboratory of Biosensors and Bioelectronics, Institute for Biomedical Engineering, ETH Zurich, 8092 Zurich, Switzerland; 4 Clinical Virology, University Hospital Basel, 4031 Basel, Switzerland; 5 Transplantation & Clinical Virology, Department Biomedicine, University of Basel, 4031 Basel, Switzerland; 6 Infectious Diseases & Hospital Epidemiology, University Hospital Basel, 4031 Basel, Switzerland; 7 Botnar Research Centre for Child Health, 4051 Basel, Switzerland

**Keywords:** SARS-CoV-2, COVID-19, PCR, sensitivity, specificity, self-swabbing, buccal

## Abstract

This study compared SARS-CoV-2 RNA loads at different anatomical sites, and the impact of self-swabbing and food intake. Adult symptomatic patients with SARS-CoV-2 or non-SARS-CoV-2 respiratory tract infection were included between 2021 and 2022. Patients performed a nasal and buccal swab before a professionally collected nasopharyngeal/oropharyngeal swab (NOPS). Buccal swabs were collected fasting and after breakfast in a subgroup of patients. SARS-CoV-2 RNA loads were determined by nucleic acid testing. Swabbing convenience was evaluated using a survey. The median age of 199 patients was 54 years (interquartile range 38–68); 42% were female and 52% tested positive for SARS-CoV-2. The majority of patients (70%) were hospitalized. The mean SARS-CoV-2 RNA load was 6.6 log_10_ copies/mL (standard deviation (SD), ±1.5), 5.6 log_10_ copies/mL (SD ± 1.9), and 3.4 log_10_ copies/mL (SD ± 1.9) in the professionally collected NOPS, and self-collected nasal and buccal swabs, respectively (*p* < 0.0001). Sensitivity was 96.1% (95% CI 90.4–98.9) and 75.3% (95% CI 63.9–81.8) for the nasal and buccal swabs, respectively. After food intake, SARS-CoV-2 RNA load decreased (*p* = 0.0006). Buccal swabbing was the preferred sampling procedure for the patients. In conclusion, NOPS yielded the highest SARS-CoV-2 RNA loads. Nasal self-swabbing emerged as a reliable alternative in contrast to buccal swabs. If buccal swabs are used, they should be performed before food intake.

## 1. Introduction

Severe acute respiratory syndrome coronavirus 2 (SARS-CoV-2) causing coronavirus disease 2019 (COVID-19), is responsible for a major health crisis still generating repercussions in the fourth year after its emergence. Testing for SARS-CoV-2 remained one of the cornerstones to identify infected individuals early. Diagnostic testing allowed the breaking of chains of infection and enabled timely management of risk-stratified treatment, infection control measures, and contact tracing [1,2]. The gold standard testing technique defined by the World Health Organization (WHO) [3,4] is nucleic acid testing, preferably in a quantitative format (QNAT). This molecular assay is characterized by a high sensitivity and specificity [4]. However, the reliable performance of diagnostic QNATs is a resource-consuming and costly process, requiring laboratory set-up and professionally trained persons to process and collect the specimens. Although the burden on laboratories and resources was partly alleviated by rapid antigen testing and appropriate self-testing, QNAT remained the reference test for critical decision making in the clinics [5,6].

The performance of a nasopharyngeal or the combination of a nasopharyngeal with a pharyngeal swab (NOPS) collected by health care personnel was recommended by the WHO [5]. Nasal or mid-turbinate self-testing has been increasingly performed during the pandemic, and has been shown to give reliable results compared to professional collection of NOPS [7,8]. However, nasopharyngeal, nasal, and pharyngeal swabbing frequently cause discomfort or pain and may result in unwillingness to test, nose bleeding, and a higher risk of virus transmission to the health care personnel due to provoked sneezing and coughing [9,10]. Subsequently, saliva was increasingly used for large-scale testing, although it showed a lower sensitivity (approx. 85%) than NOPS [4,11] and was more difficult and time-consuming to process compared to swabs [12]. Due to its less invasive and more comfortable collection technique, it was accepted as a testing technique, in particular in children or elderly people, and in the setting of repetitive routine testing. Nevertheless, the easiest swabbing method might be buccal swabbing. It causes the least uncomfortable sensations, is easy to learn, and may be performed at home [13,14]. However, the SARS-CoV-2 RNA loads of buccal swabbing procedures may be influenced by additional factors such as enzymatic degradation, hygiene procedures, or food intake, leading to a lower test sensitivity observed in this specimen [15,16]. In previous studies, SARS-CoV-2 RNA loads have rarely been measured with quantitative test methods, but semi-quantitative QNAT systems (e.g., Roche cobas^®^ system) or rapid antigen testing have been used to assess test performances.

This study aimed to compare SARS-CoV-2 RNA loads between different anatomical sites (nasal, buccal, nasopharyngeal/pharyngeal) and between professionally and self-collected swabs using QNAT. Furthermore, we aimed to assess the impact of food intake on RNA load in self-collected buccal swabs and patients’ satisfaction according to the swabbing method and location.

## 2. Materials and Methods

### 2.1. Study Design

This project was a prospective, observational, single-center, cohort study at the University Hospital Basel, a 750-bed tertiary care center in Switzerland. Patients were enrolled between January 2021 and December 2022. The study was approved by the Ethics Committee of Northwest and Central Switzerland (EKNZ Project-ID: 2020-02260) and conducted in accordance with the Declaration of Helsinki and the principles of Good Clinical Practice. All patients provided written informed consent for participation in the study.

### 2.2. Study Population

Four different patient groups were enrolled in this study. Inclusion criteria comprised: age ≥ 18 years, and presence of one of the following: (1) at least one of the influenza-like illness (ILI) and/or COVID-19 symptoms and laboratory confirmed SARS-CoV-2 infection (by a positive SARS-CoV-2 QNAT result); (2) at least one of the ILI and/or COVID-19 symptoms, but laboratory confirmed SARS-CoV-2 negative (by a negative SARS-CoV-2 QNAT result) and detection of another virus causing respiratory tract infections; (3) at least one of the ILI and/or COVID-19 symptoms but laboratory confirmed SARS-CoV-2 negative (by a negative SARS-CoV-2 QNAT result) and no detection of another virus causing respiratory tract infections; or (4) outpatients presenting for SARS-CoV-2 testing at the local test center. ILI and/or COVID-19 symptoms comprised: fever, chills, cough, sore throat, rhinitis, earaches, dyspnea, headaches, dizziness, muscle/limb pain, chest pain, abdominal pain, nausea/vomiting, diarrhea, conjunctivitis, exanthema, lymphadenopathy, fatigue/weakness, dysuria, anosmia/ageusia. The onset of symptoms had to be within the previous 10 days. Exclusion criteria for all patients included previous enrolment into the current study, inability to perform a self-collected nasal or buccal swab, nose bleeding within the last 24 h, nasal surgery within the last 2 weeks, and acute facial trauma. For the routine SARS-CoV-2 testing and confirmation of infection, a professional NOPS was collected and analyzed via QNAT on the cobas^®^ platform. A positive QNAT result was defined by a cycle threshold (Ct) value of <40.

### 2.3. Study Procedures and Sample Collection

After obtaining informed consent, written and visual but no oral instructions for self-collection of nasal (both nostrils) and buccal swabs were given to the patient (Figures S1–S3). The study physician was always present when self-swabbing was performed. A subgroup of patients who tested positive for SARS-CoV-2 (*n* = 24) was asked to collect two buccal swabs: the first fasting in the morning and before teeth brushing and the second after breakfast.

Nasal self-swabbing was performed by inserting the swab 2–3 cm into one nostril, twisting it five times, and leaving it there for ten seconds before the procedure was repeated with the same swab in the other nostril. Buccal self-swabbing was performed by swabbing five times on the inside of the cheek on both sides. For each swabbing location (nasal and buccal), a separate swab was used. After the swabbing procedure, which was not supported by the study physician, each swab was put into a separate tube containing 1.2 mL of viral culture medium. Subsequently, a NOPS was collected by a trained study physician. Both swabs were pooled into one tube containing 1.2 mL of viral culture medium.

Viral culture media (0.86× Hanks balanced salt solution (Gibco, Thermo Fisher Scientific, Waltham, MA, USA, 14060040), pH 7.4 adjusted by sodium bicarbonate solution) containing the swabs were directly processed to perform the QNAT or stored (max. 4 h) on ice or in the refrigerator (2–8 °C) before processing. After vortexing for 30 s, aliquots of 250 μL were prepared. The RNA loads of SARS-CoV-2 were determined by QNAT targeting the S-gene encoding a surface protein, the spike protein, of the virus. SARS-CoV-2 RNA loads were expressed in log_10_ copies/mL (c/mL) [17]. A SARS-CoV-2 RNA load of <1000 c/mL was defined as a negative test result. Syndromic panel testing for respiratory infections other than SARS-CoV-2 such as influenza A/B or respiratory syncytial virus was performed with QNAT using the Biofire^®^ respiratory panel (biomerieux, Marcy-l’Étoile, France), GeneXpert^®^ for co-testing of SARS-CoV-2, Influenza A, Influenza B and RSV (Cepheid, Sunnyvale, CA, USA) or the cobas^®^ platform for co-testing of SARS-CoV-2, Influenza A and Influenza B (Roche, Rotkreuz, Switzerland) [17]. After the swabbing procedures, convenience and acceptance of the swabbing methods were assessed using a 15-item questionnaire. Regarding the questionnaire, answers about the swabbing convenience were summarized into four categories: uncomplicated, acceptable, uncomfortable, painful, and not tolerable, which was merged into the fourth category.

Patient data were prospectively collected and managed using Research Electronic Data Capture (REDCap^®^ 14.2.1) hosted at the University Hospital Basel. Routine clinical variables were extracted from the patient’s records of the hospital information system and were entered in an electronic case report form in REDCap^®^ [18,19].

### 2.4. Statistical Analysis

The Mann–Whitney U-test, the Chi Square test, and Fisher’s exact test were used where appropriate. Data on SARS-CoV-2 RNA load were analyzed by a mixed-effects model with post hoc Tukey adjustment or by paired-sample *t*-test to compare means. Variable selection for characteristics of study participants was based on biological plausibility and/or demonstrated associations in the literature. For the correlation testing, Spearman correlation was used. Results were considered statistically significant if the *p*-value was less than 0.05. To calculate the diagnostic accuracy of the swabbing procedure, sensitivity, specificity, positive and negative likelihood ratios, and positive and negative predictive values were calculated with their 95% confidence interval (CI). Furthermore, sensitivity was calculated fasting compared to after breakfast for a sub-group of patients. The reference standard method was the NOPS collected by a trained study physician. All analyses were performed with SPSS Version 28 (IBM SPSS Statistics for Windows. Armonk, NY, USA) and GraphPad Prism 9.0.0 (GraphPad Software, San Diego, CA, USA).

## 3. Results

### 3.1. Demographic and Clinical Characteristics

**Figure 1:**
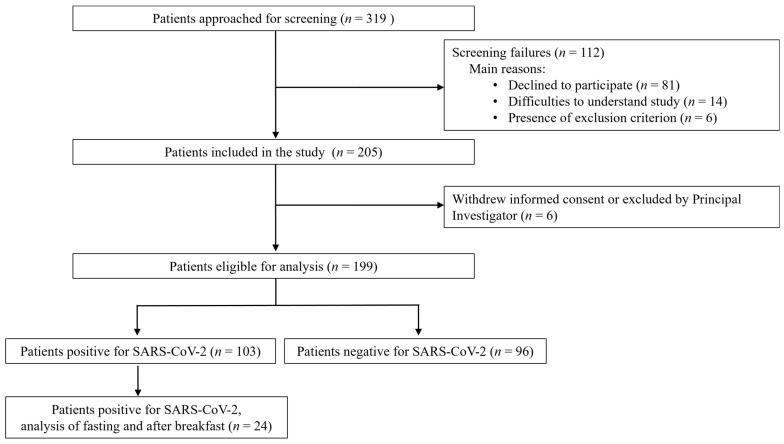
Flow chart. Two patients were excluded at the investigator’s discretion due to intolerance of nasopharyngeal swabbing, and due to replication of SARS-CoV-2 only in the lower respiratory tract at time of inclusion. SARS-CoV-2, severe acute respiratory syndrome coronavirus 2.

A total of 205 patients was enrolled in this study, and 199 patients completed the study (Figure 1).

Outpatients were most frequently recruited at the test center (60/61, 98%). Four patients withdrew their consent, and two patients were excluded at the investigator’s discretion because of difficulties in performing the self-swabbing, and SARS-CoV-2 replication confined to the lower respiratory tract only. The median age was 54 years (interquartile range (IQR) 38–68), 42% (83/199) were female, and 70% were hospitalized (139/199). Overall, 103/199 patients (52%) were tested positive for SARS-CoV-2. The median time from symptom onset to the swabbing was 4 days (IQR 2–7). The majority of patients had either one (*n* = 45, 23%) or no comorbidity (*n* = 86, 43%). In both groups of patients tested negative or positive for SARS-CoV-2, the main comorbidities were arterial hypertension (21.9% and 23.3%, respectively), cardiovascular disease (15.6% and 23.3%, respectively), and diabetes mellitus (14.6% and 19.4%, respectively) (Table 1).

Symptoms present among patients who tested positive for SARS-CoV-2 are presented in Table S1.

### 3.2. Comparison of SARS-CoV-2 RNA Load According to the Swabbing Location

**Figure 2:**
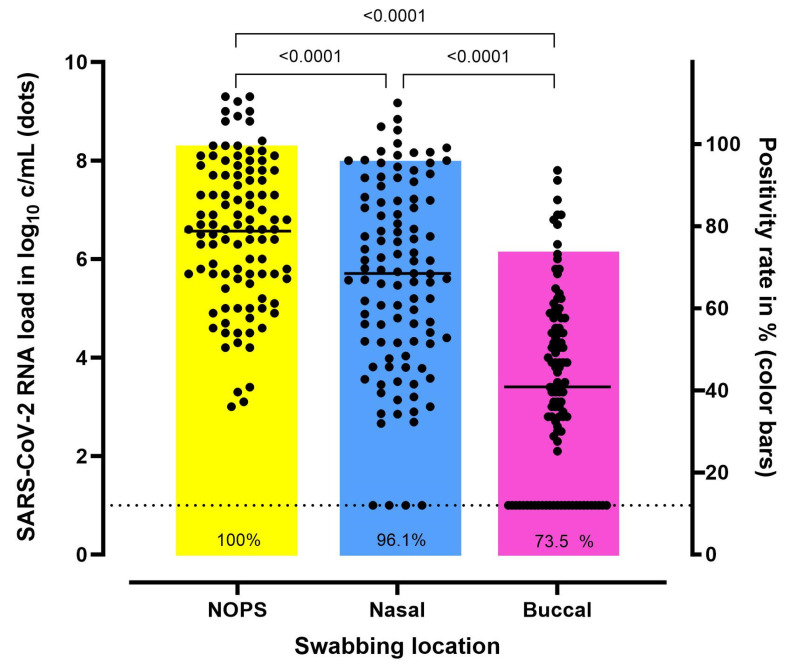
Comparison of the SARS-CoV-2 RNA load in professionally collected nasopharyngeal/oropharyngeal (NOPS) swabs and in self-collected nasal and buccal swabs (*n* = 103). In patients who performed two buccal swabs (*n* = 24), the result of the first (fasting) swab was included. Horizontal lines represent the mean of the SARS-CoV-2 RNA load in log_10_. The RNA loads of SARS-CoV-2 were determined by a quantitative nucleic acid amplification test targeting the S-gene encoding the spike protein, of the virus. SARS-CoV-2 RNA load was analyzed by a mixed-effects model with post hoc Tukey adjustment. Dashed line: limit of detection. NOPS, nasopharyngeal/oropharyngeal swab; SARS-CoV-2, severe acute respiratory syndrome coronavirus 2.

In total, 103 SARS-CoV-2 positive-tested patients were included. The RNA load in one of the 103 buccal samples could not be determined. The mean SARS-CoV-2 RNA load was 6.6 log_10_ c/mL (standard deviation (SD), ±1.5) in the professionally collected NOPS and 5.6 log_10_ c/mL (SD, ±1.9) and 3.4 log_10_ c/mL (SD, ±1.9) in the self-collected nasal and buccal swabs, respectively, *p* < 0.0001 (Figure 2).

In a Bland–Altman plot, the difference between SARS-CoV-2 RNA loads in NOPS and nasal swabs is displayed (Figure S4). Compared to NOPS, 4/103 (4%) and 27/102 (26%) of the nasal and buccal swabs were false negative, respectively. The SARS-CoV-2 RNA load in the nasal and the NOPS correlated negatively with the symptom duration (r = −0.52, *p* < 0.001). A similar result was observed with regard to buccal swabs (r = −0.335, *p* < 0.001).

### 3.3. Diagnostic Accuracy

Table 2 displays the diagnostic value of the different swabbing locations. Nasal and buccal self-swabbing showed a sensitivity of 96.1% (95% CI: 90.4–98.9) and 75.3% (95% CI: 63.9–81.8), respectively. Specificity was high at both anatomical sites (nasal 100% (95% CI 96.2–100, buccal 99.0% (94.3–100))). The majority of false-negative test results was observed in the group of self-collected buccal swabs.

### 3.4. The Impact of Food Intake on Buccal SARS-CoV-2 RNA Load

**Figure 3:**
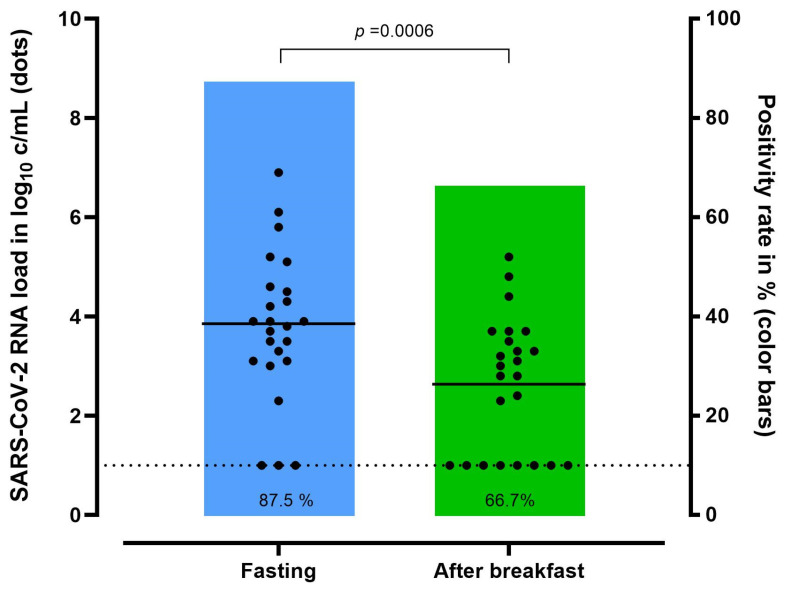
Impact of food intake on the SARS-CoV-2 RNA load in self-collected buccal swabs before and after breakfast, and according to positivity rate compared to professionally performed nasopharyngeal/pharyngeal test (*n* = 24). Horizontal lines represent the mean of the SARS-CoV-2 RNA load in log_10_. Dashed line: limit of detection. SARS-CoV-2 RNA load was analyzed by a paired-sample t-test to compare means. SARS-CoV-2, severe acute respiratory syndrome coronavirus 2.

In 24/103 patients (23%) who tested positive for SARS-CoV-2, two buccal self-swabs were performed before (fasting) and after breakfast. The mean SARS-CoV-2 RNA load was 3.8 log_10_ c/mL (SD, ±1.5) before and 2.6 log_10_ c/mL (SD, ±1.3) after breakfast (*p* = 0.0006) (Figure 3).

Fasting swabbing demonstrated a sensitivity of 87.5% (95% CI: 67.6–97.3), while a decrease in sensitivity was seen after food intake to 66.7% (95% CI: 44.7–84.4).

### 3.5. Patients’ Convenience Related to the Collection Method and Anatomical Site

**Figure 4:**
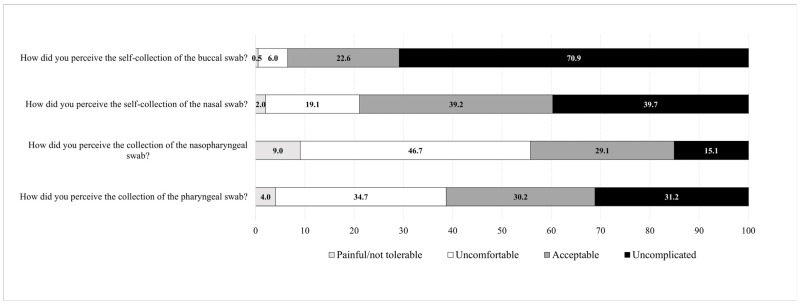
Convenience and acceptance related to self-collected swabs and the swabbing location as assessed by a questionnaire (*n* = 199, 100%).

The questionnaire was completed by all 199 patients. The majority of patients (191/199, 96%) stated that the self-instructions for nasal and buccal self-swabbing were easy to follow. Overall, 64% (127/199) of the patients had never performed a nasal swab, and 89% (178/199) had never performed a buccal swab before participating in the study. The professionally collected NOPS were painful/not tolerable or caused discomfort to 56% (111/199) and 39% (77/199) of the patients, respectively. Self-collected nasal and buccal swabs were stated to cause pain or discomfort in 21% (42/199) and 7% (13/199) of the patients, respectively (Figure 4).

The nasal and buccal self-swabbing were easy to perform for 91% (182/199) and 97% (192/199) of the patients, respectively. Furthermore, the great majority of patients was confident to perform nasal or buccal self-testing at home (191/199, 96%, and 192/199, 96%, respectively). Most patients (131/199, 66%) would prefer self-swabbing over being swabbed by a professional healthcare worker.

## 4. Discussion

In the present study, we evaluated SARS-CoV-2 RT-PCR testing at different locations in a cohort of 199 patients, and demonstrated that NOPS yielded the highest SARS-CoV-2 RNA loads. Nasal self-swabbing evolved as a reliable alternative to professionally collected NOPS. Buccal swabs had a significantly lower sensitivity compared to NOPS and nasal swabbing, but were the most convenient swabbing procedure for the patient. SARS-CoV-2 RNA loads decreased significantly after food intake.

The SARS-CoV-2 RNA load differed significantly between NOPS, nasal, and buccal swabbing, demonstrating a sensitivity of 96.1% for nasal and 73.5% for buccal self-swabbing for the detection of SARS-CoV-2. Despite a slightly lower SARS-CoV-2 RNA load observed in nasal swabs compared to NOPS, the high sensitivity of 96.1% of self-collected nasal swabs underscores the reliability of this swabbing location, which has already been shown previously [4,20,21,22]. Consequently, nasal swabbing entered international guideline recommendations as a reliable alternative for professionally collected nasopharyngeal swabs [5,23]. Furthermore, self-collected nasal swabs for SARS-CoV-2 are easy to perform, and are highly accepted [8,24,25]. Interestingly, the sensitivity of the nasal swabs was slightly higher in the present study compared to previously published studies, stating a sensitivity of approximately 85% for professionally collected or self-collected nasal swabs [4,26,27,28]. This observed difference may be related to the sensitive quantitative testing method used in the present study compared to semi-quantitative techniques or rapid antigen testing used in previous studies [4,27,29,30]. Although it has been shown that semi-quantitative techniques correlate qualitatively and quantitatively, the sensitivity of the QNAT used in the present study might be slightly higher [17]. In line, Teo et al. demonstrated that not only different testing techniques, but also the use of different RT-PCR kits impacts the sensitivity of the test results [31]. Furthermore, the majority of patients were hospitalized and were included early during the course of the disease, therefore representing a sicker patient population with a possibly higher viral load. Additionally, the introduction of public self-testing strategies may have led to an improved quality of the self-swabbing procedure and a higher sensitivity.

In the present study, the sensitivity of buccal swabs was 73.5% and, therefore, significantly lower than the sensitivity of nasal swabs. In previous studies, their performance was even poorer (sensitivity of oral swabs (rapid antigen tests): 9% [32], 18% [13], and 32% [15]) (sensitivity of buccal swabs (semi-quantitative QNAT): 20.8% [33], 65.4% [9]. 56.7% [34]). The distinctly higher sensitivity in the present study may be associated with the very sensitive testing method used, but also the patient population included, as the majority of patients were symptomatic and the median time between symptom onset and inclusion in the study was only 4 days, resulting in high SARS-CoV-2 RNA loads at the time of testing. Furthermore, the differences in sensitivity of oral or buccal swabbing in previous studies might be related to the instructions patients were given before swabbing, ranging from fasting 30 min before testing [13,15] to additional coughing before sample collection [15] or no instructions at all [33]. Optimal patient instructions may be crucial, especially when collecting buccal or saliva samples. If correctly instructed and performed, saliva samples may even yield a comparable or higher sensitivity compared to NOPS [31]. In line with this, the sensitivity of buccal swabs increased significantly in the present study when the patients were optimally instructed, i.e., to perform the buccal swabbing fasting.

The higher SARS-CoV-2 RNA load in the fasting samples may be related to viral particles accumulating in the oropharynx and mouth during nighttime, resulting in higher oral virus concentrations in the morning [35]. During food intake, more saliva is produced, which leads to a dilution of viral particles in the mouth. Furthermore, a large number of viral particles are probably swallowed [36]. Saliva is rich in RNA degrading enzymes and potential PCR inhibitors, which may break down the viral RNA, and reduce the yield even of the very sensitive QNAT testing. A similar impact on viral load has been presented when saliva samples are collected after mouthwashing [37].

Overall, despite the higher sensitivity of buccal self-swabbing observed in the present study compared to previously published studies, it cannot be recommended as a reliable alternative to the professionally collected NOPS or the self-collected nasal swab due to the high rate of false-negative results, which is in line with previous studies [9,32]. If buccal swabs or oral specimens are used for SARS-CoV-2 testing, it is recommended to collect them fasting in the morning before food intake, drinking, or brushing teeth, to increase the overall sensitivity. Importantly, the positivity rate would further decrease when other, less sensitive test methods such as rapid antigen tests are used [38], and when the patient is not instructed correctly.

In the survey conducted in the present study, the majority of patients (56%) judged professionally collected NOPS as painful/not tolerable or causing discomfort. Conversely, self-collected nasal and buccal swabs were rated in regard to the same inconveniences with only 21% and 7%, respectively. Accordingly, two-thirds of the patients (66%) would prefer self-swabbing over being swabbed by a professional healthcare worker, which is in line with previous studies [26,39,40].

The presented study has several limitations. The COVID-19 patients were all inpatients, representing a more severely ill patient population. There was a selection bias, as patients who participated in the study were more likely to be interested in COVID-19-related testing in general and, hence, the performance of self-swabbing might be better compared to the general population. Additionally, as a member of the study team was always present during the self-swabbing procedure, the participants may have followed the instructions more closely. As the questionnaire was not answered anonymously, but in the presence of the study team, the answers may have been biased, as patients may have felt that a socially accepted answer should be provided.

However, the results of our study may not solely be applicable for SARS-CoV-2 infection, but also for the confirmation of other viral respiratory pathogens, and, hence, may be relevant for testing strategies in future pandemics. In line, Jung et al. demonstrated the superiority of NOPS for detecting non-SARS-CoV-2 respiratory viruses, but concluded that nasal swabs and saliva samples may represent alternatives to NOPS [28], which is in accordance with our findings.

In conclusion, SARS-CoV-2 RNA load differed significantly between professionally collected NOPS and self-collected nasal and buccal swabs. Self-performed nasal swabs proved to be a reliable diagnostic method in symptomatic COVID-19 patients. The practice of nasal swab collection is supported by patients favoring self-swabbing over professional swabbing. Conversely, buccal swabbing cannot be recommended as an alternative swabbing method due to the high rate of false negative results. Food intake impacts significantly on the oral SARS-CoV-2 RNA load. Therefore, if buccal swabbing or other oral specimens are used, they should be collected during fasting in the morning. As convenience, acceptance, and performance of nasal self-swabbing is high, it should remain a cornerstone of any SARS-CoV-2 testing strategy. These results may inform testing strategies in future pandemics.

## Figures and Tables

**Table 1:** Baseline characteristics of the study participants. Data are presented as count (percentages) or median (interquartile range).

Variable	COVID-19 Patients *n* = 103	Non-COVID-19 Patients *n* = 96	All Patients *n* = 199
Sex: female	40 (38.8)	43 (44.8)	83 (42)
Age in years, median	55 (43–71)	51 (34–65)	54 (38–68)
Length of hospital stay in days	6 (3–8)	8 (5–13)	6 (4–9)
Inpatients	101 (98.1)	37 (38.5)	138 (69)
Main diagnosis for admission:			
COVID-19	92 (89)	-	92 (46)
Congestive heart failure	1 (1)	1 (1)	2 (1)
COPD exacerbation	-	8 (8)	8 (4)
Pneumonia other than COVID-19	-	8 (8)	8 (4)
Other	8 (8)	21 (22)	29 (15)
No admission	2 (2)	58 (60)	60 (30)
Comorbidities:			
Arterial hypertension	24 (23.3)	21 (21.9)	45 (23)
Cardiovascular disease	24 (23.3)	15 (15.6)	39 (20)
Diabetes mellitus	20 (19.4)	14 (14.6)	34 (17)
Chronic kidney disease	12 (11.7)	10 (10.4)	22 (11)
Asthma	11 (10.7)	11 (11.5)	22 (11)
Hematological disease/cancer	10 (7.5)	20 (20.8)	30 (15)
Chronic lung disease	9 (9.7)	18 (18.8)	27 (14)
Pathogen identified (other than SARS-CoV-2):			
Coronavirus HKU1	-	1 (1)	1 (1)
Coronavirus NL63	-	4 (4)	4 (2)
Coronavirus OC43	-	1 (1)	1 (1)
Human Rhinovirus/Enterovirus	-	7 (7)	7 (4)
Parainfluenza virus 3	-	2 (2)	2 (1)
Respiratory syncytial virus	-	1 (1)	1 (1)
Vaccinated against SARS-CoV-2:			
Yes	38 (36.9)	19 (19.8)	57 (29)
No	32 (31.1)	16 (16.7)	48 (24)
Unknown	33 (32.0)	61(63.5)	94 (47)

Abbreviations: COVID-19: coronavirus disease 2019; COPD: chronic obstructive pulmonary disease; SARS-CoV-2: severe acute respiratory syndrome coronavirus type 2.

**Table 2:** Diagnostic value of self-collected nasal and buccal swabs for the detection of SARS-CoV-2 RNA.

Swabbing Location	*n*	Sensitivity (95% CI)	Specificity (95% CI)	PLR (95% CI)	NLR (95% CI)	PPV (95% CI)	NPV (95% CI)
Nasal	103	96.1% (90.4–98.9)	100% (96.2–100)	-	0.04 (0–0.10)	100% (96.3–100)	96.0% (90.2–98.4)
Buccal	102 *	75.5% (63.9–81.8)	99.0% (94.3–100.0)	70.6 (10.0–497.8)	0.27 (0.2–0.4)	98.7% (91.4–99.8)	77.9% (71.8–83.0)

* Analysis of one buccal sample was not possible. CI, confidence interval; PLR, positive likelihood ratio; NLR, negative likelihood ratio; PPV, positive predictive value; NPV, negative predictive value.

## Data Availability

The datasets generated are available from the corresponding author on reasonable request (requiring ethics approval).

## Supplementary Materials

The following supporting information can be downloaded at: https://www.mdpi.com/article/10.3390/microorganisms12030591/s1, Figure S1: Instruction file 1 for the self-collection of nasal and buccal swabs; Figure S2: Instructions for the self-collection of nasal swabs; Figure S3: Instructions for the self-collection of buccal swabs; Figure S4: Bland–Altman plot showing the differences of SARS-CoV-2 RNA load between professionally collected nasopharyngeal/oropharyngeal swabs and nasal swabs. NOPS, nasopharyngeal/oropharyngeal swab; SARS-CoV-2, severe acute respiratory syndrome coronavirus 2. Table S1: Symptoms and clinical signs of the patients tested positive for SARS-CoV-2 (*n* = 103). Data are presented as count (percentages) or median (interquartile range).
