# Supplementary material for: Impact of Swabbing Location, Self-Swabbing, and Food Intake on SARS-CoV-2 RNA Detection
Source: Microorganisms. 2024 Mar 15;12(3):591. doi: 10.3390/microorganisms12030591 (PMC10976265; doi:10.3390/microorganisms12030591)
Supplement: Supplementary file 1 [file microorganisms-12-00591-s001.zip › microorganisms-2914818-supplementary.pdf]

Supplement

Supplement Figure S1. Instruction file 1 for the self-collection of nasal and buccal swabs.

Materials overview:

Beaker 1:

- Plastic tube 1 (filled with liquid)
- Swab 1

Beaker 2:

- Plastic tube 2 (filled with liquid)
- Swab 2

1 Waste collection beaker

1 Disinfectant

2 Instructions

1 Questionnaire

Please note:

When taking the swabs, please ensure that you proceed carefully and that you do not cause any pain to yourself.

Supplement Figure S2. Instructions for the self-collection of nasal swabs.

1. Nasal swabbing

1. Disinfect your hands.
2. Take **beaker 1.**
3. Take **plastic tube 1**, unscrew the lid, and put it back in the beaker with the lid open.
4. Take **swab 1** and open the packaging at the yellow mark.
5. Take the swab out of the packaging without touching the cotton and keep it in your hand.
6. Insert the swab **2-3 cm straight** (horizontally) into **one** nostril until you feel resistance. Turn it **5x** and leave it there for **10 seconds.**
7. Repeat the procedure **with the same swab in the other** nostril.
8. Place the swab with the cotton facing downwards into the **plastic tube 1**, bend it at the red predetermined breaking point, and close the tube with the lid.

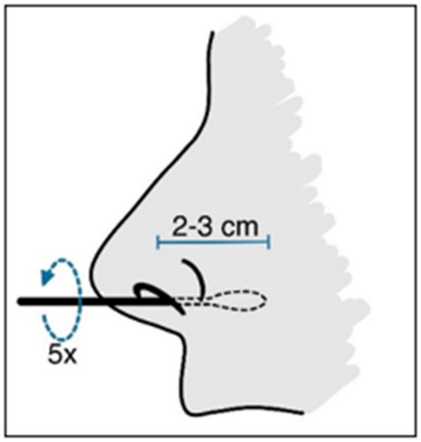

Supplement Figure S3. Instructions for the self-collection of buccal swabs.

2. Buccal swabbing

1. Take **beaker 2.**

2. Take **plastic tube 2**, unscrew the lid, and put it back in the beaker with the lid open.

3. Take **swab 2** and open the packaging at the green mark.

4. Take the swab out of the packaging without touching the cotton and keep it in your hand.

5. Open your mouth and rub the swab firmly over the inside of your cheeks - both sides **5x** each.

6. Place the swab with the cotton facing downwards into the **plastic tube 2**, bend it at the red predetermined breaking point, and close the tube with the lid.

7. Disinfect your hands.

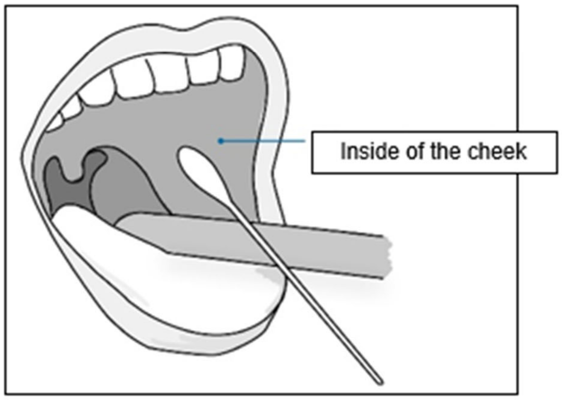

Supplement Figure S4. Bland-Altman plot showing the differences of SARS-CoV-2 RNA load between professionally collected nasopharyngeal/pharyngeal swabs and nasal swabs. NOPS, nasopharyngeal/oropharyngeal swab; SARS-CoV-2, severe acute respiratory syndrome coronavirus 2.

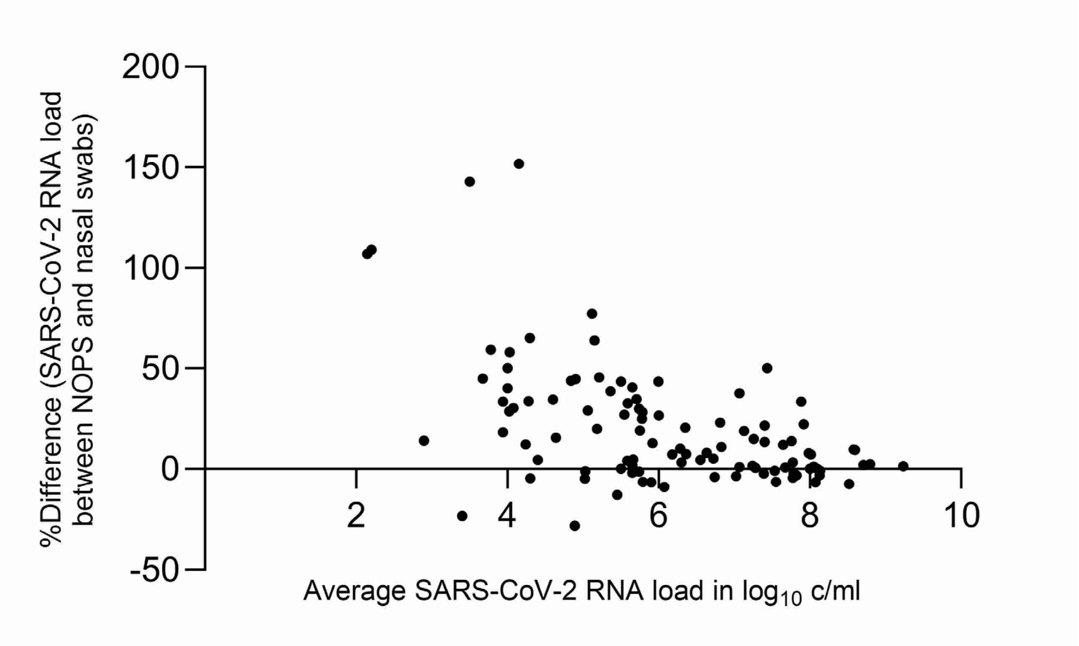

Supplement Table S1. Symptoms and clinical signs of the patients tested positive for SARS-CoV-2 (n=103). Data are presented as count (percentages) or median (interquartile range).

Variable	Patients positive for SARS-CoV-2, n=103
Symptoms	
Fever	71 (68.9)
Cough	68 (66.0)
Dyspnea	42 (40.8)
Fatigue/ Weakness	42 (40.8)
Headache	32 (31.1)
Muscle/limb pain	27 (26.2)
Diarrhea	21 (20.4)
Nausea/ Vomiting	20 (19.4)
Sore throat	19 (18.4)
Chest pain	19 (18.4)
Rhinitis	13 (12.6)
Chills	11 (10.7)
Anosmia	10 (9.7)
Other	8 (7.8)
Abdominal pain	7 (6.8)
Dizziness	6 (5.8)
Dysurea	2 (1.9)
Earaches	1 (1.0)
Lymphadenopathy	1 (1.0)
Number of symptoms per patient	4 (3-5)
Respiratory rate ≥ 22/min	42 (40.8)
Peripheral oxygen saturation	94% (92−96)
Peripheral oxygen saturation < 92%	18 (17.5)
Need of supplemental oxygen	36 (35.0)
Reasons for testing	
Symptoms	99 (96.0)
Infection control screening	2 (1.9)
Outbreak	1 (1.0)
At own request	1 (1.0)
